# 

**Published:** 1918-01-19

**Authors:** 


Rates of Subscription: Twelve months, 6/6; Six months, 4/-; three months, 2/-, post free to any address in the United Kingdom. Abroad, Twelve months, 8/8; Six months, 5/-; Three months, 2/6, post free. **THE HOSPITAL "Index" to Volume LXII., April 1917 to September 1917, is now ready, price 2½d. per copy, post free.** Address The Manager, The Hospital, "The Hospital" Building, 28/29 Southampton Street, Strand, London, W.C. 2.

### Accommodation in Civil Hospitals.

THE terms of reference to the Committee recently appointed for the survey of institutional accommodation and facilities of treatment in Great Britain are: "To ascertain the accommodation in civil and other hospitals (not being Poor-Law institutions) for the treatment of sick persons and the facilities available for medical and surgical treatment and care, other than that given by general practitioners, in Great Britain; to consider, regard being had both to the needs of disabled men discharged from naval or military service, and to those of the civilian population, the extent to which, and the means by which, such accommodation and facilities should, if necessary, be increased, and to report thereon at an early date."

The members of the section of the Committee which will deal with England and Wales are:—Sir A. G. Boscawen, Chairman; the Hon. Sir A. Stanley, Red Cross; Sir A. Newsholme, Local Government Board; Dr. J. Smith-Whitaker, National Health Insurance Commission, England; Dr. H. Meredith Richards, National Health Insurance Commission, Wales; Sir George Bullock and Colonel Webb, R.A.M.C., War Office; Sir J. Collie, Sir A. Pearce Gould, and Mr. C. F. A. Hore, Assistant-Secretary to the Ministry of Pensions. It is proposed to appoint a separate section of the Committee to deal with Scotland, and the desirability of appointing a member to represent voluntary hospitals is being considered.

### Personalia.

Captain James Rafter, M.B., R.A.M.C., awarded the Military Cross, was house surgeon of the Liverpool David Lewis Northern Hospital before he enlisted at the outbreak of the war.

Mr. George Dean, the steward of Lambeth Infirmary, has resigned his position, Dr. Baly certifying that from his knowledge of the steward's health during the past few years he was of opinion that he was permanently unfit to perform his duties. Mr. Dean's resignation was accepted, and it was decided to add five years to his term of service for the purpose of computing his allowance under the Poor-Law Officers Superannuation Act, 1896.

Captain Wilfred Gibbons, R.A.M.C. (T.) who died at the end of December after only a few days' illness, was stationed at the 5th Northern General Hospital, in the town of Leicester, where he was a well-known medical practitioner. Educated at Charterhouse, he qualified at Edinburgh in 1896, and graduated M.D. at the same University in 1900. He held the post of house surgeon at the Edinburgh Royal Infirmary, and represented his medical school in Association Football. He had held his post at the Base Hospital since November 1914. His widow and two children survive him. He was an ex-president of the Leicestershire and Rutland Branch of the British Medical Association.

In the list we published last week (January 12, p. 311) of appointments to the Order of the British Empire, Mr. A. P. Hughes Gibb, O.B.E., was described as "late assistant secretary to the Seamen's Hospital, now private secretary to the Food Controller." We are informed that Mr. Hughes Gibb is still assistant secretary to the Seamen's Hospital, but he is doing war work as private secretary to the Food Controller. Mr. Fletcher Toomer is meantime acting assistant secretary at Greenwich.

### Matrons' and Sisters' Appointments.

CITY FEVER HOSPITAL, COVENTRY.—Miss Mabel Rowley has been appointed night sister. She was trained at Bolton Infirmary and Monsall Fever Hospital.

COTTAGE HOSPITAL, WELLS, NORFOLK.—Miss Ruth Jones has been appointed matron. She was trained at the Birmingham General Hospital, and has been matron at the Erith Cottage Hospital, Stamford and Rutland General Infirmary, and at the Guest Hospital, Dudley.

CRUMPSALL INFIRMARY.—Miss M. C. Owen has been appointed ward sister at this infirmary, where she was trained.

GENERAL HOSPITAL, TUNBRIDGE WELLS. — Miss C. M. Spencer has been appointed sister. She was trained at the Gloucestershire Royal Infirmary, and has since been sister at the Royal West Sussex Hospital, Chichester.

HIGHFIELD MILITARY HOSPITAL, LIVERPOOL.—Miss Lillian Shimmin has been re-appointed sister. She was trained at Highfield Infirmary, later being sister there. Since the outbreak of war she has been nursing in France.

HOSPITAL ST. CROSS, RUGBY.—Miss Isabella Richardson has been appointed sister. She was trained at the Chelmsford General Hospital, and has been night sister at the General Hospital, Merthyr, and charge nurse at the Accident Hospital, Walker. She has also done private nursing.

ILKLEY CORONATION COTTAGE HOSPITAL.—Miss Mary Allan has been appointed matron. She was trained at the Huddersfield Royal Infirmary, and has been sister at the Surgical Hospital, Stockton-on-Tees, night superintendent at the Coventry and Warwickshire Hospital, Coventry, and ward sister at the Preston Royal Infirmary.

ODIHAM COTTAGE HOSPITAL, WINCHFIELD.—Miss E. M. Martin has been appointed matron. She was trained at Wandsworth Union Infirmary, and has been matron at Swanage Cottage Hospital.

PRINCESS CHRISTIAN HOSPITAL, WEYMOUTH.—Miss Rachel Hoare has been appointed sister of the Women's and Children's Ward. She was trained at the London Temperance Hospital, and has since been surgical sister at Clandon Military Hospital, Guildford, and sister at Paddington Green Children's Hospital.

ROYAL EYE AND EAR HOSPITAL, BRADFORD.—Miss Jean Miller has been appointed sister. She was trained at the Royal Infirmary, Liverpool, has been sister at Shrewsbury Eye Hospital and at the Ear and Throat Hospital, Birmingham, and has been sister-in-charge of operating theatre in hospitals in Serbia and Greece.

ROYAL GWENT HOSPITAL, NEWPORT, MON.—Miss Hilda Vine has been appointed matron. She was trained at the East London Hospital for Children and at Guy's Hospital, at which latter institution she was afterwards sister for five years and hospital housekeeper for one year. She was then appointed Examiner for the Incorporated Society of Trained Masseuses. She has done military nursing under Queen Alexandra's Imperial Nursing Service Reserve, served for one year on H.M.S. *Asturias*, and has been theatre and block sister at the War Hospital, Reading.

THE INFIRMARY, ISLEWORTH.—Miss Selina Mary Brown has been appointed ward sister. She was trained at the East Dulwich Infirmary, and has been sister at the Royal British Orphan Schools, Slough, and at the Children's Infirmary, West Norwood.

VICTORIA COTTAGE HOSPITAL, GUERNSEY.—Miss Lilian Mary Vernon Forsdick has been appointed sister. She was trained at Leicester Royal Infirmary, where she was later sister, and has been night sister at the War Hospital, Redhill.

WAR HOSPITAL, REDHILL, SURREY.—Miss Evelyn Hilton has been appointed night sister. She was trained at Charing Cross Hospital, has been matron at the Throat and Ear Hospital, Brighton, and has done military nursing as sister in India.

**NOTE.—Particulars of Appointments for insertion in this column will be welcomed.**

### Gifts and Bequests.

JOHANNESBURG has sent £4,000 for the Royal Flying Corps Hospital.

MR. WILLIAM WRIGLEY, of Bent House, Meltham, left £100 to the Huddersfield Infirmary.

MISS MARY JENKINS, the headmistress of a local elementary school, left £100 to the Swansea Hospital.

THE Chelsea Hospital for Women has received £500, bequeathed by the late Miss Catherine Lloyd for investment.

MRS. CLARA WIGLEY, of "Redcliffe," Mapperley Road, Nottingham, left £100 to the Nottingham Hospital for Women.

THE Hon. Jane Maria Duncombe, of Westwood Mount, Scarborough, left £100 to the Scarborough Hospital and Dispensary.

A SWANSEA fishmonger, Mr. A. Andrews, has given £2,000 to the Swansea Hospital to endow a bed in memory of his mother.

MISS ELEANOR ERLE, of Calverley Park, Tunbridge Wells, bequeathed £3,000 to Ockenden Convalescent Home, Torquay.

MR. GEORGE GILL, of Walsall, left £1,000 each to the Walsall and District Hospital and to the Walsall Victoria Nursing Institution.

MR. SAMUEL WAKEFIELD, of Pudgate, Lancs, left £100 to the Warrington Infirmary and £50 to the Warrington and District Nurses' Association.

MR. WILLIAM GLADSTONE, formerly Rector of Bo'ness Academy, left £500 to Glasgow Royal Infirmary and £500 to Edinburgh Royal Infirmary.

THE Chelsea Hospital for Women and the West London Hospital have each received a grant of £1,000 from the trustees of the Annie Zunz bequest.

THE Royal Devon and Exeter Hospital has received a donation of £500 from its President, Sir E. Channing Wills, towards the appeal to wipe off the debit balance.

ALDERMAN WILLIAM SANDERSON, of Sunderland, bequeathed to the Sunderland Orphan Asylum £500 of Midland Railway stock and £250 of Great Central Railway stock.

